# Discordance between Glucose Management Indicator and Glycated Hemoglobin in a Pediatric Cohort with Type 1 Diabetes: A Real-World Study

**DOI:** 10.3390/children11020210

**Published:** 2024-02-06

**Authors:** Simone Foti Randazzese, Bruno Bombaci, Serena Costantino, Ylenia Giorgianni, Fortunato Lombardo, Giuseppina Salzano

**Affiliations:** Department of Human Pathology in Adult and Developmental Age “Gaetano Barresi”, University of Messina, Via Consolare Valeria 1, 98124 Messina, Italy; simone.foti.92@gmail.com (S.F.R.); brunobombaci@gmail.com (B.B.); serenacostantino29@gmail.com (S.C.); ylenia.g18@gmail.com (Y.G.); fortunato.lombardo@unime.it (F.L.)

**Keywords:** adolescent, automated insulin delivery, blood count, children, continuous glucose monitoring, glycemic variability, glycosylation, hybrid closed loop, insulin, time in range

## Abstract

The introduction of continuous glucose monitoring (CGM) systems in clinical practice has allowed a more detailed picture of the intra- and interdaily glycemic fluctuations of individuals with type 1 diabetes (T1D). However, CGM-measured glucose control indicators may be occasionally inaccurate. This study aims to assess the discrepancy between the glucose management indicator (GMI) and glycated hemoglobin (HbA1c) (Δ_GMI-HbA1c_) within a cohort of children and adolescents with T1D, exploring its correlation with other CGM metrics and blood count parameters. In this single-center, cross-sectional study, we gathered demographic and clinical data, including blood count parameters, HbA1c values, and CGM metrics, from 128 pediatric subjects with T1D (43% female; mean age, 13.4 ± 3.6 years). Our findings revealed higher levels of the coefficient of variation (CV) (*p* < 0.001) and time above range > 250 mg/dL (*p* = 0.033) among subjects with Δ_GMI-HbA1c_ > 0.3%. No association was observed between blood count parameters and Δ_GMI-HbA1c_. In conclusion, despite the advancements and the widespread adoption of CGM systems, HbA1c remains an essential parameter for the assessment of glycemic control, especially in individuals with suboptimal metabolic control and extreme glycemic variability.

## 1. Introduction

Type 1 diabetes (T1D) is a chronic disease of considerable public health significance, exhibiting an escalating incidence rate in recent decades [1,2]. Due to its peculiar absolute insulin deficiency, T1D requires lifelong management involving insulin replacement therapy through either multiple daily injections consisting of administering long-acting and rapid-acting insulin via subcutaneous injections or continuous subcutaneous insulin infusion (CSII) through insulin pumps.

In recent decades, significant advances have been made in diabetes care through the implementation and development of technological devices [3,4]. Insulin pumps, which provide a continuous basal insulin rate and deliver bolus doses as required (e.g., before meals or to correct hyperglycemia), simulate physiological basal and personalized insulin secretion, showing improved glycemic outcomes compared to multiple daily injection therapy [5]. The introduction of predictive low-glucose suspend systems, a result of linking insulin pumps to continuous glucose monitoring (CGM) systems to suspend insulin delivery in the event of impending low glucose levels, has further enhanced the safety and quality of life for individuals with T1D [6].

More recently, automated insulin delivery systems have gained approval for clinical use. These devices incorporate algorithms that automatically adjust the insulin delivery rate to maintain as much blood glucose levels within the physiological range as much as possible. Two generations of automated insulin delivery systems are currently used in clinical practice: the first generation, known as a hybrid closed loop, solely offers automated adjustments of the basal insulin delivery rate to achieve a predetermined sensor glucose target, while the second generation, or an advanced hybrid closed loop, adds the possibility of delivering automatic correction boluses [4].

T1D can significantly affect the quality of life for both children and their caregivers. The ongoing demands of monitoring blood glucose and administering insulin, as well as the associated risks of hypoglycemia, hyperglycemia, and potential chronic complications, pose substantial challenges that may disrupt routine daily activities [7,8].

To optimize the management of T1D, a careful and proactive monitoring of blood glucose levels is fundamental, offering valuable insights that guide not only the precise adjustment of insulin dosages but also essential lifestyle modifications. Glycated hemoglobin (HbA1c) plays a pivotal role as a marker for the assessment of longitudinal glucose control in individuals with diabetes, both in clinical practice and experimental studies [9]. This marker is the end product of a glycosylation process involving the covalent binding between glucose and the N-terminal valine of the hemoglobin β chain. Considering the typical lifespan of red blood cells, which is approximately 120 days, the HbA1c value is influenced by the concentration of blood glucose over the preceding 8 to 12 weeks [10]. Therefore, HbA1c offers a comprehensive evaluation of long-term glycemic control in contrast to daily self-monitoring of blood glucose [11] and facilitates appropriate treatment adjustments [12]. Furthermore, extensive research has demonstrated a strong correlation between HbA1c levels and the long-term risk of complications, including micro- and macrovascular diseases [13,14,15]. Consequently, international guidelines recommend regular quarterly HbA1c measurements for all children and adolescents with T1D [11].

CGM systems have now become the standard of monitoring for children and adolescents with T1D [16]. These systems, by measuring the glucose concentration of the interstitial fluid, provide real-time insights into blood glucose levels, as well as information on trends in average daily glucose, time spent within the target range, and glucose variability [17]. There are currently two types of CGM systems in use: real-time CGM (rtCGM), which automatically detects glucose levels continuously throughout the day and night, typically at intervals ranging from 1 to 5 min, and intermittently scanned CGM (isCGM), which measures the interstitial glucose concentration only upon the user’s request, using a dedicated reader [18]. Numerous randomized clinical trials and real-world studies focusing on pediatric populations with T1D have consistently demonstrated the positive impact of CGM use on diabetes management [19].

With the increasing use of CGM devices, CGM metrics are now considered reliable indicators, alongside HbA1c, for assessing glucose control in subjects with T1D. In daily clinical practice, particular emphasis is given to metrics such as the time spent within, above, and below the target glycemic range. These metrics offer immediate insights into glycemic trends, facilitating the prompt identification of the duration and extent of hypo- and hyperglycemia. Another crucial metric is the coefficient of variation (CV), a percentage value representing sensor glucose variation within a specified time interval. The CV is directly correlated with sensor glucose standard deviation and is inversely correlated with mean sensor glucose, making it an important parameter to consider in assessing glycemic variability [20]. In addition, the glucose management indicator (GMI) serves as an estimated HbA1c value, calculated using CGM data from a selected period [21]. Formerly referred to as estimated A1C (eA1C), this metric is derived through a formula established from a regression line, plotted with mean blood glucose concentration points on the x axis and contemporaneously measured HbA1c values on the y axis [21]. GMI is widely recognized as a useful indicator and a valuable substitute in instances where laboratory HbA1c is unavailable. However, HbA1c and GMI values may be often discordant, generating confusion and frustration and requiring careful consideration when the two parameters are compared in daily clinical practice [22].

This study aims to assess the discordance between GMI and HbA1c in a cohort of children and adolescents with T1D, exploring its association with other CGM metrics and blood count parameters.

## 2. Materials and Methods

In this cross-sectional observational study, we recruited children and adolescents with T1D attending the pediatric diabetes outpatient service at our tertiary-care center (University Hospital of Messina) from May 2022 to April 2023. All study participants, along with their parents, received comprehensive information and provided informed consent as part of the study protocol. The study was conducted according to good clinical practice and in compliance with the Declaration of Helsinki and its successive amendments. Ethical committee approval was not required, as the study used anonymized and unidentifiable data routinely collected at our diabetes center.

Inclusion criteria were age < 18 years, a diagnosis of T1D according to the latest ISPAD guidelines [23], and use of CGM for at least three months prior to recruitment. Exclusion criteria included daily sensor use <70%, the presence of partial remission phase according to the Hvidovre study definition [24], uncontrolled concomitant diseases, chronic use of paracetamol or other drugs known to interfere with the accuracy of some glycemic sensors, and chronic therapy with corticosteroids or other drugs capable of interfering with blood glucose levels and/or blood count.

At the time of enrolment, demographic, anamnestic, and clinical data were collected, including biological sex, age, duration of disease, ethnicity, comorbidities, auxological parameters, type of CGM system (isCGM or rtCGM), type of insulin treatment (multiple daily injections, sensor-augmented pump, hybrid closed loop, or advanced hybrid closed loop), and the most recent blood count parameters (erythrocyte count, hemoglobin, hematocrit, mean corpuscular volume, mean corpuscular hemoglobin, mean corpuscular hemoglobin concentration, red cell distribution width coefficient of variation, leukocyte, lymphocytes, neutrophils, monocytes, eosinophil and basophil count, and platelet count). HbA1c measurements were performed via capillary fingerstick using a DCA Vantage Analyzer (Siemens**^®^**, New York, NY, USA). The method was not based on high-performance liquid chromatography (HPLC). All HbA1c measurements were conducted on the day of recruitment, and subjects were unaware of their inclusion in the study until that day.

CGM data from the 15-day period preceding enrolment for rtCGM users and from the 30-day period before enrolment for isCGM users were retrospectively collected for each subject from specific Web cloud platforms (i.e., Carelink Pro, Libreview, Dexcom Clarity, Glooko platforms). The following CGM metrics were calculated: mean sensor glucose, percentage of time between 70 and 180 mg/dL (TIR), percentage of time between 180 and 250 mg/dL (TAR_Level1_), percentage of time above 250 mg/dL (TAR_Level2_), percentage of time between 54 and 70 mg/dL (TBR_Level1_), percentage of time below 54 mg/dL (TBR_Level2_), GMI, and CV expressed in percentage.

Intraindividual differences between GMI and HbA1c (Δ_GMI-HbA1c_) were calculated for each subject, and the cohort was stratified into 3 subgroups based on Δ_GMI-HbA1c_ values: Δ_GMI-HbA1c_ ≤ −0.3%, −0.3% < Δ_GMI-HbA1c_ ≤ 0.3%, and Δ_GMI-HbA1c_ > 0.3%. These thresholds were adopted arbitrarily in order to obtain three homogeneous subgroups.

### Statistical Analysis

Numerical data were expressed as mean and standard deviation, and categorical variables as absolute frequency and percentage. The Kolmogorov–Smirnov test showed the normal distribution of the numerical variables, so a parametric approach was used. Comparisons between subgroups based on Δ_GMI-HbA1c_ values (Δ_GMI-HbA1c_ ≤ −0.3%, −0.3% < Δ_GMI-HbA1c_ ≤ 0.3%, and Δ_GMI-HbA1c_ > 0.3%) were performed using the ANOVA test for numerical parameters.

Univariate and multivariate linear regression models were employed to identify significant predictors of Δ_GMI-HbA1c_ values. Age, biological sex, duration of diabetes, presence of comorbidities, BMI Z-score, mean sensor glucose, erythrocyte count, hemoglobin, hematocrit, mean corpuscular volume, mean corpuscular hemoglobin, mean corpuscular hemoglobin concentration, red cell distribution width coefficient of variation, leukocytes, sensor use, CV, TIR, TAR_Level1_, TAR_Level2_, TBR_Level1_, and TBR_Level2_ were considered covariates in these models.

Data analysis was performed using Microsoft Excel version 2023 and Statistical Package for Social Sciences (SPSS) version 22.0.

A *p* value < 0.05 was considered statistically significant.

## 3. Results

Our study population consisted of a cohort of 128 subjects, with a slight male prevalence (57%). Upon enrolment, the mean age of participants was 13.4 ± 3.6 years, the mean duration of diabetes was 5.8 ± 9 years, and the body mass index (BMI) Z score was 0.5 ± 0.91. Most of individuals (99.2%) were of Caucasian ethnicity. Comorbidities were present in 21.1% of participants, with celiac disease being the most frequently reported (9.4%).

Among the cohort, 84.4% used rtCGM systems, while the remaining 15.6% adopted isCGM. Regarding treatment strategies, 21.1% of participants were on multiple daily injection therapy, while sensor-augmented pumps, predictive low-glucose suspend, hybrid closed-loop, and advanced hybrid closed-loop systems were used by 14.8%, 8.6%, 6.3%, and 49.2% of individuals, respectively. In the entire study cohort, the mean HbA1c was 6.7 ± 0.7%, the GMI was 7.0 ± 0.6%, and TIR had a mean value of 69.2 ± 12.4%. Demographic, anamnestic, clinical, and anthropometric data of the study participants are summarized in Table 1.

Based on their Δ_GMI-HbA1c_, all subjects were stratified into three subgroups: 22.7% with Δ_GMI-HbA1c_ ≤ −0.3%, 35.2% with −0.3% < Δ_GMI-HbA1c_ ≤ 0.3%, and 42.2% with Δ_GMI-HbA1c_ > 0.3%. These subgroups were homogeneous for age (*p* = 0.723) and BMI Z-score (*p* = 0.532). Additionally, individuals on MDI therapy were equally distributed among the three subgroups (*p* = 0.242).

### 3.1. Comparison between Subgroups with Different Δ_GMI-HbA1c_ Values

As shown in Table 2, a comparison of glucose control indicators among subgroups stratified according to Δ_GMI-HbA1c_ values revealed a higher CV among subjects with Δ_GMI-HbA1c_ > 0.3% compared to those with Δ_GMI-HbA1c_ ≤ −0.3% and −0.3% < Δ_GMI-HbA1c_ ≤ 0.3% (38 ± 6.2% vs. 35.2 ± 4.2% vs. 34.5 ± 4.4%; *p* < 0.001). Similarly, the subgroup with Δ_GMI-HbA1c_ > 0.3% showed higher TAR_Level2_ values (9 ± 8.9% vs. 6.1 ± 5.8% vs. 5.5 ± 4.6%; *p* = 0.033). Additionally, mean sensor glucose levels were lower in the group with Δ_GMI-HbA1c_ > 0.3% (*p* < 0.001). No other significant differences were detected between the TIR, TAR_Level1_, TBR_Level1_, and TBR_Level2_ of the three subgroups.

When considering blood count parameters, no significant differences were observed between the erythrocyte count, hemoglobin, hematocrit, mean corpuscular volume, mean corpuscular hemoglobin, mean corpuscular hemoglobin concentration, red cell distribution width coefficient of variation, leukocyte, lymphocytes, neutrophils, monocytes, eosinophil and basophil count, and platelet count of different Δ_GMI-HbA1c_ subgroups.

After performing a separated analysis of subjects using MDI therapy, we confirmed a significant difference in mean sensor glucose (*p* < 0.001) among the three subgroups with different Δ_GMI-HbA1c_ values, mirroring a trend similar to that observed in the general population.

### 3.2. Clinical Predictors of Δ_GMI-HbA1c_

Univariate linear regression analysis revealed a negative association between Δ_GMI-HbA1c_ values and mean sensor glucose (B = −0.014, *p* < 0.001), along with a positive association with TAR_Level2_ values (B = 0.012, *p* = 0.045). These associations were further validated through multivariate regression analysis (Table 3).

## 4. Discussion

In the contemporary landscape marked by the widespread adoption of CGM systems in the management of T1D, HbA1c still stands as the primary indicator of glucose control in clinical practice and is adopted as the glycemic outcome in the majority of real-world studies and clinical trials evaluating treatment effectiveness [11,25,26,27]. The utility and reliability of this marker as a predictor of long-term complications in individuals with T1D were initially established by the Diabetes Control and Complications Trial (DCCT). This multicenter, randomized, controlled clinical trial involving 1441 subjects revealed reduced risks of diabetic retinopathy, nephropathy, neuropathy, and macrovascular disease in the cohort receiving intensive insulin treatment compared to standard therapy [28]. Based on this solid evidence, international guidelines have been established for children and adolescents with T1D, setting a target HbA1c below 7% to define satisfactory glucose control. In selected individuals deemed at high risk of severe hypoglycemia, this target can be extended to 7.5% [11].

Nevertheless, HbA1c presents some major limitations. First, due to its direct correlation with average blood glucose levels, this marker lacks comprehensive information about glucose variability [29]. Additionally, conditions such as anemia, hemoglobinopathies, iron deficiency, and pregnancy can impact HbA1c levels [11]. Therefore, the use of GMI, a CGM-measured estimated value of HbA1c, finds application in various clinical settings, especially in the presence of factors influencing HbA1c [11], and in the context of remote T1D management through telehealth medicine [30], as observed during the COVID-19 pandemic [31].

Supporting the widespread use of GMI as a metric of longitudinal glucose control, previous studies have demonstrated a strong relationship between GMI and HbA1c [30]. However, our findings revealed a discordance of at least 0.3% between HbA1c and GMI in over half of the subjects in a pediatric cohort. This substantial discordance has been previously reported in adult populations [32]. Similarly, a real-world analysis of CGM data of 805 pediatric subjects conducted by Piona et al. showed discordance between these two indicators in more than a third of cases [22].

Besides its application as a marker for assessing long-term glucose control during the follow-up of individuals with diabetes, the measurement of HbA1c also serves as a diagnostic tool. According to American Diabetes Association guidelines, an HbA1c level ≥ 6.5% is considered a diagnostic criterion for diabetes [1]. However, considering the frequent discordance observed between HbA1c and GMI values, caution is advised against using the latter for diagnostic purposes.

The main finding of our cross-sectional study was that subjects with GMI at least 0.3% higher than the HbA1c value exhibited greater glycemic variability and spent more time with sensor glucose levels exceeding 250 mg/dL. This result suggests that GMI should be carefully interpreted in subjects with brittle glycemic control and frequent glycemic fluctuations. The use of CGM systems has recently provided valuable insights into short-term glucose variability patterns in T1D subjects, with emerging evidence suggesting glycemic variability as an independent risk factor for diabetes-related long-term complications and severe hypoglycemia [33]. Several cross-sectional studies examining CGM data in individuals with T1D have identified a correlation between increased glycemic variability metrics and the occurrence of retinopathy, elevated albuminuria, neuropathy, and coronary artery calcification, regardless of HbA1c levels [34]. Despite the use of advanced technologies, including second-generation automated insulin delivery systems [35,36], achieving recommended CV targets [37] remains challenging, particularly in the pediatric population [38]. This trend is confirmed by the mean CV of our study cohort, slightly exceeding the 36% target recommended by the International Consensus on CGM data interpretation [37]. Several factors influencing glycemic variability in children and adolescents with T1D have been identified, including physical activity [39], pubertal status [40], meal composition [41], irregular sleep patterns [42], and the presence of insulin-induced lipodystrophies [43].

Surprisingly, our study found lower mean glucose levels in the subgroup with Δ_GMI-HbA1c_ > 0.3%. However, given the evidence of longer time spent with sensor glucose levels exceeding 250 mg/dL in the same subgroup, this finding should be ascribed to high glycemic variability rather than overall better glycemic control.

HbA1c is a glycemic control marker strictly dependent on the interaction between the concentration of blood glucose and the lifespan of the red blood cells. This feature affects its accuracy in the case of concurrent medical conditions disrupting the physiological turnover of erythrocytes. Specifically, conditions that diminish the lifespan of erythrocytes and subsequently increase their turnover, such as chronic hemorrhage, hemolytic anemia, and hemoglobinopathies, tend to lower HbA1c by reducing hemoglobin’s exposure to the glycosylation process [44]. Cystic fibrosis, a genetic disease often associated with cystic fibrosis-related diabetes, frequently leads to a reduced erythrocyte lifespan, rendering HbA1c an unreliable indicator of glucose control in this category of subjects [45]. Conversely, conditions that decrease erythrocyte turnover, such as macrocytic anemia and iron deficiency, can result in falsely elevated HbA1c levels [46].

Given these peculiarities of HbA1c, a discordance between HbA1c and GMI values should be expected in all subjects with the aforementioned conditions. However, our results showed that none of the examined hematologic parameters were significantly associated with the discrepancy between GMI and HbA1c. Our findings align with previous literature data, suggesting no influence of hemoglobin concentration on GMI-HbA1c discordance in pediatric subjects [22]. These findings suggest that, despite the acknowledged relationship between blood count parameters, HbA1c, and glycemic control [47], this discordance should be attributed to other underlying causes.

Despite several points of strength, including the sample size and the novelty of the research, the main limitation of our study is the single-center design. Specifically, the variance in baseline glycemic control among children and adolescents with T1D from different regions, coupled with the predominantly Caucasian ethnic background of our cohort, restrains the generalizability of our findings. This underscores the necessity of similar analyses among individuals from diverse geographic areas and non-Caucasian populations.

## 5. Conclusions

In our cohort of children and adolescents with T1D using CGM systems, a substantial percentage of individuals showed discordant HbA1c and GMI values. HbA1c remains an essential parameter for assessing glycemic control, and GMI should be interpreted cautiously in subjects with suboptimal metabolic control and extreme glycemic variability. To confirm these results, further studies involving populations mixed for ethnicity are needed.

## Figures and Tables

**Table 1:** Summary of demographic, anamnestic, clinical, and anthropometric data of study participants.

Variables	Frequency and Mean ± SDS
**Number of subjects**	128
**Age (**years**)**	13.4 ± 3.6
**Duration of diabetes (ys)**	5.8 ± 9
**Age at onset (ys)**	7.6 ± 3.8
**Ethnicity** Caucasian Others	127 (99.2%) 1 (0.8%)
**Biological sex** Male Female	73 (57%) 55 (43%)
**Comorbidities** Yes No	27 (21.1%) 101 (78.9%)
**BMI Z-score**	0.5 ± 0.91
**HbA1C (%)**	6.7 ± 0.7
**GMI (%)**	7.0 ± 0.6
**Sensor use (%)**	92.3 ± 9.5
**Glucose monitoring system** isCGM rtCGM	20 (15.6%) 108 (84.4%)
**Insulin treatment type** MDI SAP PLGS HCL AHCL	27 (21.1%) 19 (14.8%) 11 (8.6%) 8 (6.3%) 63 (49.2%)

AHCL: advanced hybrid closed loop; BMI: body mass index; GMI: glucose management indicator; HbA1C: glycated hemoglobin; HCL: hybrid closed loop; isCGM: intermittently scanned continuous glucose monitoring; MDI: multiple daily injections; PLGS: predictive low-glucose suspend; rtCGM: real-time continuous glucose monitoring; SAP: sensor-augmented pump.

**Table 2:** Comparison of glucose control indicators and blood count parameters between subgroups stratified according to Δ_GMI-HbA1c_ values.

	Δ ≤ −0.3% (*n* = 29)	−0.3% < Δ ≤ 0.3% (*n* = 45)	Δ > 0.3% (*n* = 54)	*p*	Cohort
**Age (years)**	12.8 ± 3.2	13.2 ± 3.4	13.7 ± 3.8	0.724	13.4 ± 3.6
**BMI (Z-score)**	0.66 ± 1	0.56 ± 0.99	0.5 ± 0.83	0.532	0.5 ± 0.91
**Mean sensor glucose (mg/dL)**	172.7 ± 20.6	150.8 ± 13.4	134.7 ± 17.8	<0.001 *	147.9 ± 25
**CV (%)**	35.2 ± 4.2	34.5 ± 4.4	38 ± 6.2	<0.001 *	36.2 ± 5.4
**TIR (%)**	71.1 ± 11.1	70.6 ± 10.5	67.8 ± 14.1	0.407	69.2 ± 12.4
**TAR_Level1_ (%)**	19.7 ± 6.2	20.9 ± 7.3	19 ± 7.2	0.421	20.1 ± 7
**TAR_Level2_ (%)**	6.1 ± 5.8	5.5 ± 4.6	9 ± 8.9	0.033 *	7.3 ± 7.1
**TBR_Level1_ (%)**	2.5 ± 1.8	2.4 ± 2.4	3 ± 2.2	0.276	2.7 ± 2.2
**TBR_Level2_ (%)**	0.5 ± 0.9	0.5 ± 0.8	0.8 ± 0.8	0.24	0.6 ± 0.8
**RBC (million/mm^3^)**	5.04 ± 4.63	4.97 ± 3.41	5.10 ± 5.53	0.391	5.03 ± 4.69
**Hb (g/dL)**	14 ± 1.3	14.1 ± 1.1	14.4 ± 1.5	0.294	14.2 ± 1.3
**Hct (%)**	40.8 ± 3.2	41.4 ± 3.1	42 ± 4	0.593	41.5 ± 3.5
**MCV (fL)**	81.6 ± 5.4	83.6 ± 4.6	83.3 ± 7.6	0.388	83.1 ± 6.2
**MCH (pg)**	28 ± 2.3	28.5 ± 1.8	28.4 ± 2.7	0.656	28.4 ± 2.3
**MCHC (%)**	34.1 ± 1.4	33.9 ± 0.7	33.9 ± 1	0.674	33.9 ± 1
**RDW-CV (%)**	13.4 ± 0.7	13.3 ± 0.7	13.3 ± 0.9	0.711	13.3 ± 0.8
**WBC (n/mm^3^)**	6652.7 ± 1518	6614.9 ± 1725.8	6784 ± 1861.3	0.881	6694.8 ± 1729.7
**PLT (n/mm^3^)**	285,379.3 ± 59,613	271,555 ± 53,927	283,962 ± 67,026	0.520	279,921 ± 60,864

BMI: body mass index; CV: coefficient of variation; GMI: glucose management indicator; Hb: hemoglobin; Hct: hematocrit; HbA1c: glycated hemoglobin; MCH: mean corpuscular hemoglobin; MCHC: mean corpuscular hemoglobin concentration; MCV: mean corpuscular volume; RBC: red blood cell; RDW-CV: red cell distribution width—coefficient of variation; TAR_Level1_: time above the range of 180–250 mg/dL; TAR_Level2_: time > 250 mg/dL; TBR _Level2_: time < 54 mg/dL; TBR_Level1_: time below the range of 54–70 mg/dL; TIR: time within the range of 70–180 mg/dL; WBC: white blood cell; PLT: platelet. * Significant *p* value.

**Table 3:** Results of univariate and multivariate linear regression models for Δ_GMI-HbA1c_.

**Univariate Linear Regression**			
**Variable**	***B***	**95% CI**	***p*** **-Value**
Biological sex (male)	−0.029	−0.231; 0.174	0.781
Age	0.014	−0.014; 0.042	0.310
Duration of diabetes	0.005	−0.022; 0.032	0.699
Comorbidities	−0.001	−0.247; 0.245	0.995
BMI Z score	−0.052	−0.160; 0.056	0.340
Mean sensor glucose	−0.014	−0.017; −0.011	<0.001 *
RBC	0.001	−0.001; 0.003	0.456
Hemoglobin	0.043	−0.032; 0.118	0.258
Hct	0.021	−0.007; 0.049	0.131
MCV	0.008	−0.008; 0.024	0.302
MCH	0.013	−0.030; 0.056	0.546
MCHC	−0.043	−0.141; 0.054	0.380
RDW-CV	−0.054	−0.179; 0.071	0.395
WBC	0.000	−0.006; 0.005	0.867
Sensor use	0.007	−0.005; 0.018	0.250
CV	0.014	−0.005; 0.032	0.140
TIR	−0.004	−0.013; 0.004	0.276
TAR_Level1_	−0.001	−0.015; 0.013	0.908
TAR_Level2_	0.012	0.002; 0.026	0.045 *
TBR_Level1_	0.009	−0.036; 0.054	0.697
TBR_Level2_	0.045	−0.072; 0.162	0.445
**Multivariate Linear Regression**			
**Variable**	***B***	**95% CI**	***p*** **-Value**
Biological sex (male)	−0.074	−0.206; 0.057	0.265
Age	0.010	−0.010; 0.030	0.329
Duration of diabetes	0.007	−0.010; 0.024	0.402
Comorbidities	−0.40	−0.184; 0.104	0.584
BMI Z score	−0.034	−0.091; 0.022	0.226
Mean sensor glucose	−0.023	−0.026; −0.021	<0.001 *
RBC	0.008	−0.001; 0.017	0.071
Hb	−0.210	−0.814; 0.394	0.492
Hct	−0.023	−0.197; 0.150	0.790
MCV	0.149	−0.011; 0.310	0.067
MCH	−0.291	−0.760; 0.178	0.222
MCHC	0.313	−0.169; 0.795	0.201
RDW-CV	−0.037	−0.130; 0.057	0.438
WBC	−0.001	−0.004; 0.002	0.528
Sensor use	0.003	−0.004; 0.010	0.376
CV	0.016	−0.004; 0.035	0.114
TIR	0.018	−0.042; 0.079	0.550
TAR_Level1_	0.039	−0.022; 0.100	0.206
TAR_Level2_	0.053	0.002; 0.105	0.043 *
TBR_Level1_	−0.019	−0.085; 0.048	0.578
TBR_Level2_	−0.001	−0.089; 0.087	0.984

BMI: body mass index; CV: coefficient of variation; Hb: hemoglobin; Hct: hematocrit; HbA1c: glycated hemoglobin; MCH: mean corpuscular hemoglobin; MCHC: mean corpuscular hemoglobin concentration; MCV: mean corpuscular volume; RBC: red blood cell; RDW-CV: red cell distribution width—coefficient of variation; TAR_Level1_: time above the range of 180–250 mg/dL; TAR_Level2_: time > 250 mg/dL; TBR _Level2_: time < 54 mg/dL; TBR_Level1_: time below the range of 54–70 mg/dL; TIR: time within the range of 70–180 mg/dL; WBC: white blood cell; PLT: platelet. * Significant *p* value.

## Data Availability

The data presented in this study are available on request from the corresponding author. The data are not publicly available due to the privacy of research participants.
